# Intermediate filament reorganization dynamically influences cancer cell alignment and migration

**DOI:** 10.1038/srep45152

**Published:** 2017-03-24

**Authors:** Andrew W. Holle, Melih Kalafat, Adria Sales Ramos, Thomas Seufferlein, Ralf Kemkemer, Joachim P. Spatz

**Affiliations:** 1 Max-Planck-Institute for Medical Research, Dept. of Cellular Biophysics, Stuttgart, Germany; 2 Department of Internal Medicine I, Ulm University, Ulm, Germany; 3 Reutlingen University, Reutlingen, Germany; 4 Department of Biophysical Chemistry, University of Heidelberg, Heidelberg, Germany

## Abstract

The interactions between a cancer cell and its extracellular matrix (ECM) have been the focus of an increasing amount of investigation. The role of the intermediate filament keratin in cancer has also been coming into focus of late, but more research is needed to understand how this piece fits in the puzzle of cytoskeleton-mediated invasion and metastasis. In Panc-1 invasive pancreatic cancer cells, keratin phosphorylation in conjunction with actin inhibition was found to be sufficient to reduce cell area below either treatment alone. We then analyzed intersecting keratin and actin fibers in the cytoskeleton of cyclically stretched cells and found no directional correlation. The role of keratin organization in Panc-1 cellular morphological adaptation and directed migration was then analyzed by culturing cells on cyclically stretched polydimethylsiloxane (PDMS) substrates, nanoscale grates, and rigid pillars. In general, the reorganization of the keratin cytoskeleton allows the cell to become more ‘mobile’- exhibiting faster and more directed migration and orientation in response to external stimuli. By combining keratin network perturbation with a variety of physical ECM signals, we demonstrate the interconnected nature of the architecture inside the cell and the scaffolding outside of it, and highlight the key elements facilitating cancer cell-ECM interactions.

The basis for the self-powered movement of any cell is the cytoskeleton, a cell type-specific mixture of microfilaments, microtubules, and intermediate filaments. Continual reorganization and restructuring of cytoskeletal components is essential to the survival of cells, and is crucial for a number of processes including focal adhesion turnover, morphological stability, and cell migration^1^,^2^. The actin microfilament network in particular, which has been described as the lead ‘actor’ in cell migration^1^, has been well characterized in this respect^2^,^3^,^4^,^5^,^6^, and has been shown to be sufficient for the formation of metastasis-causing invadopodia^2^. However, the interactions between this network and other cytoskeletal elements, like microtubules and intermediate filaments, have only recently been shown to be relevant^1^,^7^,^8^.

Keratin, which encompasses an intermediate filament family containing over 50 isomers split into two pH-based subtypes, plays a major role in cell-matrix interactions by stabilizing focal adhesion sites and playing a role in traction force generation^9^,^10^. Keratinocytes lacking keratin are capable of faster ECM adhesion, and are subsequently able to migrate twice as fast as wild type cells^11^. The loss of keratin isomers found in hepatoma cells is sufficient to decrease cancer cell stiffness around force-sensing focal adhesions, as well as interfere with actin-RhoA-ROCK mechanotransduction of ECM stiffness, illustrating the importance of keratin in mechanosensitive cancer biology^12^,^13^. Keratin networks are also capable of responding to local force^1^,^14^, underscoring the role keratin plays in determining the bulk stiffness of a cell^15^,^16^.

Although keratin loss does not affect actin levels or network organization^15^, there are a number of studies that have linked actin microfilaments and keratin intermediate filaments. F-actin assembly inhibition has been shown to quickly increase potentially compensatory keratin formation^17^. The molecular scaffolds stratifin and plectin have been shown to stabilize a complex of actin and keratin intermediate fibers, providing a physical linkage allowing for indirect force transmission and giving a malignant cell an arsenal of cytoskeletal components from which to initiate metastatic migration and invasion^18^,^19^.

Although the intermediate filament vimentin has been heavily implicated in the cancer invasion-conducive epithelial to mesenchymal transition (EMT)^20^,^21^, keratin has not traditionally been thought of as a key player in the mechanical basis of cancer invasion and metastasis. The lack of keratin in invadopodia^2^ and comparative dearth of methods to study intermediate filaments have reinforced this^22^. However, the use of keratin as a classical diagnostic and prognostic marker in epithelial tumors and the observed down-regulation of keratins during epithelial-mesenchymal transition (EMT) supports the notion that keratins are hardly innocent bystanders during the metastasis process^23^,^24^,^25^. There are conflicting and often cell-type specific effects of keratin knockdown and up-regulation in cancer cells, both of which have been found to curtail adhesion, migration, and invasion^25^,^26^,^27^,^28^. The ability of keratin to affect cancer cell migration and invasion is likely the result of altered phosphorylation dynamics, with effects from both increases and decreases in phosphorylation reported^29^,^30^,^31^.

Sphingosylphosphorylcholine (SPC) is a naturally occurring lipid capable of activating JNK and Erk kinases, which in turn stimulate phosphorylation of K8 and K18 keratins^31^. SPC also affects the intermediate filament vimentin by phosphorylating S71. This phosphorylation of intermediate filaments leads to an increase in perinuclear keratin and vimentin organization^32^,^33^. SPC has also been shown to enhance migration through micropores^33^,^34^ in a manner mirroring the EMT-like effects that have been observed in keratin null or keratin knockdown cells^11^,^35^. Indeed, primary cancer cells isolated from tumors also exhibited keratin organization typical of SPC treatment^36^. Cancer cells containing keratin mutants corresponding to the same phosphorylation sites that SPC targets show increased levels of cell migration and invasion^37^. As a result of this SPC-mediated priming of migratory and invasive machinery, as well as the correlation between certain types of cancer and SPC expression *in vivo*^38^ and its recent successful evaluation as a treatment target itself^39^, SPC is a valuable tool for studying the role of keratin reorganization in cancer metastasis. This value is magnified when SPC is used in conjunction with the presentation of controlled substrate topography, as the cytoskeleton is a key player in a cell’s morphological adaptation to topographical ECM cues.

In general, cancer cells are exposed to physical signals that can direct their migration and invasion, and must translate those signals into cellular responses in a process known as mechanotransduction^40^. One example of a physical signal providing this stimulation is substrate topography^41^,^42^, which is capable of directing cell polarization and migration in a process known as contact guidance^43^. This phenomenon can be stimulated and observed with the aid of microstructures such as nanogrooves^44^,^45^ and micropillars^46^,^47^. One goal of creating artificial substrates for *in vitro* cancer cell studies is to mimic the *in vivo* tumor microenvironment; by identifying and leveraging molecular mechanisms of cancer behavior *in vitro*, new treatments can be developed and improved.

It is well known that ECM stiffness is altered in the tumor microenvironment^48^, although in systems designed to test both substrate rigidity and substrate topography in the context of alignment, cancer cells were found to be far more influenced by topography than rigidity, including microgrooves and pillars^49^. Microgrooves with widths between 25 and 120 microns have been used to mimic blood vessels present in vascular tumors^50^, while nanogrooves may better mimic the arrangement of nano-scale ECM fibers in the tumor microenvironment^43^.

In addition to substrate topography, cancer cells are exposed to a wide range of mechanical forces, exerted both by the growing tumor and the pulsatile flow encountered during intravasation into the circulatory system. Cells exposed to constant strain generally align themselves in the direction of substrate stretch^51^,^52^, while those exposed to cyclic strain generally align perpendicular to the direction of substrate stretch^53^,^54^. While there is a clear physiological basis for cyclic substrate strain, it can also be used as an experimental tool to investigate if keratin phosphorylation, altered by SPC, affects the overall morphological adaptation to stretch, a question that has not been answered in the context of cancer cells. The link between cytoskeletal elements like actin and keratin and the microtopographical features a cancer cell adapts to are inherently linked, presenting a clear need for integrative analysis of both systems.

In this work, we apply SPC-mediated keratin reorganization, substrate topography, and cyclic substrate stretch to cells both independently and in combination to determine if keratin organization affects contact guidance and mechanically-induced morphological adaptations in Panc-1 epithelioid carcinoma cells. With the aid of several image analysis metrics, we have found that Panc-1 cells with altered keratin networks are capable of more dynamic force-sensitive orientation and faster, more directed migration in response to nano and microtopographical patterns. We have also made observations on the role of topographical conditions on cancer cell migration on grooved and pillared substrates, and documented optimal cyclic strain conditions for cancer cell realignment with and without keratin phosphorylation.

## Results and Discussion

### Keratin phosphorylation does not affect Panc-1 cell area but does affect actin inhibition responses

**Figure 1:**
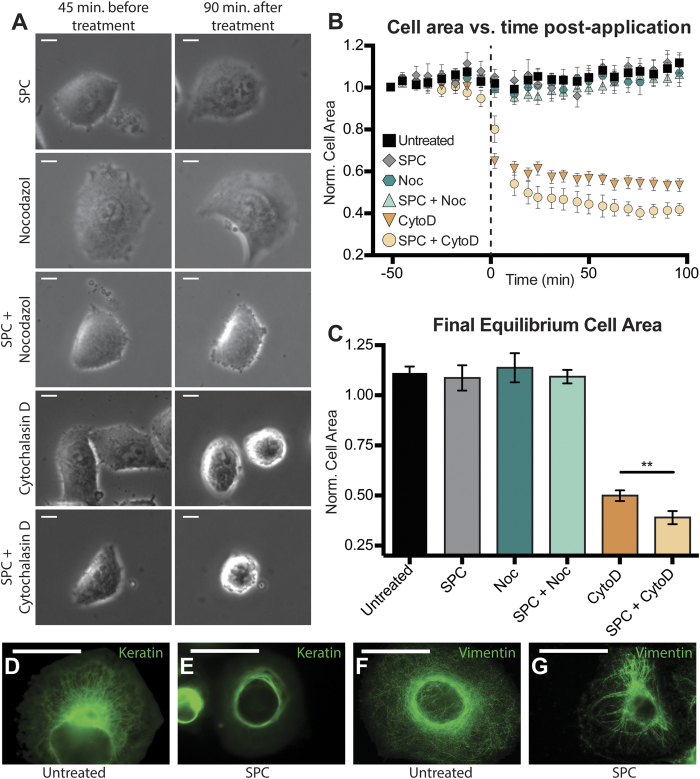
Keratin Phosphorylation and Cytoskeletal Inhibitors. (**A**) Images of single cells were taken both 45 minutes before and 90 minutes after application of inhibitors or SPC. (**B**) Average normalized cell area was calculated from the time of application to equilibrium (~90 minutes) and (**C**) final equilibrium values for normalized cell area for each treatment showed a significant difference between Cytochalasin D treatment alone and treatment in conjunction with keratin phosphorylation (**p < 0.01 by ANOVA). Untreated keratin and vimentin distribution (**D**,**F**) was altered upon application of SPC and resulted in perinuclear organization (**E**,**G**). Scale bars = 50 μm (**A**,**D**,**E**). SPC- Sphingosylphosphorylcholine, Noc- Nocodazole, CytoD- Cytochalasin D.

The effects of cytoskeletal elements on cell morphology were analyzed by treating Panc-1 cells on flat PDMS substrates with chemical inhibitors of microtubules (nocodazole), and actin (cytochalasin D) in conjunction with keratin phosphorylation (SPC). Phase contrast images were captured in 6.5 minute intervals, starting 45 minutes prior to treatment and finishing once equilibrium area was reached. Cell area at each time point was calculated from cell outlines, with the area at each time point normalized to the area of the cell immediately prior to treatment (Fig. 1A).

In untreated cells, the keratin network spans the cell from the perinuclear region to the cell membrane (Fig. 1D)^33^, while the SPC treatment concentrates the keratin almost completely around the nucleus (Fig. 1E), a pattern that is also seen in the intermediate filament vimentin (Fig. 1F,G). The absolute and normalized areas of untreated and SPC-treated cells were found to be equivalent over the course of the treatment (Fig. 1B). As with SPC treatment alone, nocodazole either alone or in parallel with SPC did not have a significant impact on cell area up to 100 minutes after treatment (Fig. 1B), allowing for the conclusion that microtubules do not play a significant role in the stabilization of cell area. Cytochalasin D treatment has previously been reported to be sufficient to drastically reduce cell area^55^, and in Panc-1 cells a 46% reduction was observed (Fig. 1C). Interestingly, this effect appears to be enhanced by SPC treatment, with parallel application resulting in a cell area decrease of as much as 59% (Fig. 1C), significantly larger (p < 0.01) than the decrease in area from Cytochalasin D alone. Based on these results, it appears that keratin and actin dynamics are interconnected- when keratin reorganizes, leaving a void in the previously enmeshed cortical region of the cell, the actin cytoskeleton may be more free to reorganize without the steric hindrance that a well defined keratin network provides. This makes keratin an attractive element for perturbation- on its own, SPC treatment will not affect simple cellular metrics like spread area, but it can free up cytoplasmic space through which the more responsive actin network can move.

### Keratin dynamics are independent of actin orientation in stretched Panc-1 cells

**Figure 2:**
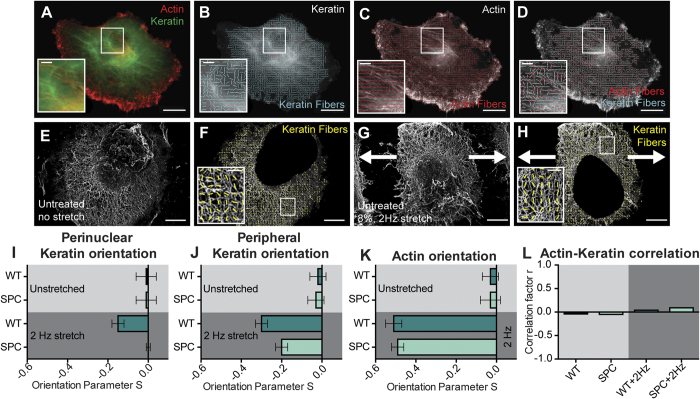
Actin and Keratin Alignment in Response to Cyclic Strain. (**A**) Immunofluorescence image example used to calculate keratin (**B**) and actin (**C**) alignment, followed by alignment correlation analysis of intersecting fibers (**D**). Electron microscopy was used to visualize peripheral keratin alignment on both unstrained and strained substrates (**E**–**H**). On unstretched substrates, keratin and actin did not display any non-random orientation (**I**–**K**, top), but upon application of 2 Hz cyclic substrate strain, all fibers realigned in a perpendicular direction, with actin responding most strongly (**I**–**K**, bottom). Despite both fibers realigning, local alignment of actin and keratin did not correlate strongly in the cyclically strained samples (**L**). Scale bars = 20 μm (**A**–**D**), 5 μm (**A**–**D** insets, **E**–**F**), 1 μm (**F**,**H** insets). WT = Untreated (**I**–**L**). Arrows indicate direction of cyclic strain (**G**,**H**).

Structural interactions between the actin and keratin networks were analyzed by comparing their spatial orientation in relation to each other. To do this, cells on flat control and cyclically stretched (2 Hz, 8%) substrates were stretched for eight hours and stained for both actin and keratin, and a custom ImageJ macro was used to correlate cytoskeletal alignment (Fig. 2A–D). Electron microscopy performed after solubilizing non-keratin cytoskeletal elements^33^ was used to compensate for the fact that immunofluorescence was not sufficient to visualize the keratin network at the periphery of the cell (Fig. 2E–H).

For cells on unstretched control PDMS substrates, the orientation of the actin fibers was randomly distributed, while cells exposed to cyclic strain exhibited fibers aligned perpendicularly to the direction of force. These alignment patterns were unaffected by SPC-mediated keratin network phosphorylation (Fig. 2K). Cells plated on unstretched substrates showed no preferential alignment of the perinuclear or peripheral keratin network, but did exhibit keratin alignment perpendicular to the direction of strain in both the periphery and the perinuclear regions, although at a lower magnitude than the actin fiber alignment (Fig. 2I,J). SPC treatment was sufficient to suppress this mechanoresponse in the perinuclear region of the cell, but in the periphery, alignment was only reduced by 33% (Fig. 2J). Higher levels of keratin alignment in the periphery of the cell during cyclic strain could be a result of the increasing role of mechanosensitive focal adhesion activity and turnover^56^.

Correlation coefficients were calculated using orientation values for any actin and keratin fibers found to intersect. From these measurements, an average correlation coefficient was calculated for each experimental condition (Fig. 2L). Although the orientation of the keratin in the stretch experiments had a slight alignment bias perpendicular to the direction of pull, no significant correlation between actin orientation and keratin orientation in fiber intersection points was found, as all values were statistically equivalent to 0. This result is surprising given the fact that both systems realign in the same direction, but it reinforces the hypothesis that the two networks behave independently and likely do not bind to each other during the realignment process. In special consideration of the fact that keratin alignment in response to cyclic strain has never been reported, only SPC-mediated keratin phosphorylation was analyzed in conjunction with substrate topography-based migration and morphology.

### Panc-1 cell orientation dynamics as a function of cyclic strain and keratin phosphorylation

**Figure 3:**
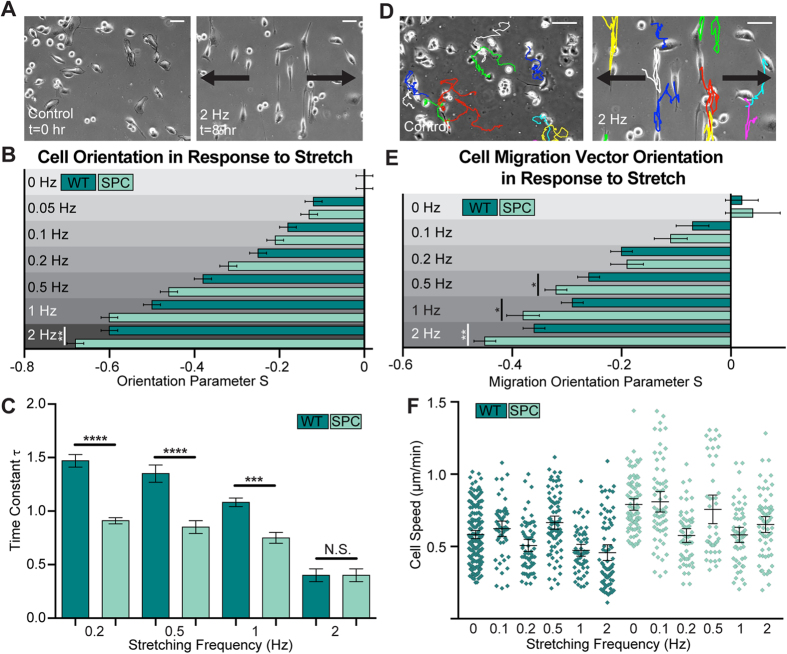
Keratin Phosphorylation and Cyclic Strain Induced Orientation and Migration. (**A**) Images of single cells were taken over the course of 8 hours during the application of various cyclic strain frequencies, with most cells aligning most efficiently perpendicular to the direction of strain at higher frequencies and with SPC treatment (**B**). Analysis of the amount of time needed to achieve orientation equilibrium revealed that SPC can augment rearrangement speed in frequencies up to 2 Hz, where the effect is lost (**C**). By tracking cells during the experiment (**D**), migration direction vectors could be calculated and analyzed in the context of alignment (**E**), with higher strain frequencies and SPC treatment both contributing to more directed migration. SPC treatment caused the cells to move faster, but strain frequency had no systemic effect on cell migration speed (**F**). Increases in cell speed were statistically significant for control substrates (p < 0.0001), 0.2 Hz (p < 0.0001), 0.5 Hz (p < 0.01), and 2 Hz substrates (p < 0.0001), all by one-way ANOVA with Tukey post hoc tests. Scale bars = 50 μm (**A**), 150 μm (**D**). WT = Untreated (**B**–**F**). Arrows indicate direction of cyclic strain (**A**,**D**).

Untreated and SPC-treated Panc-1 cells were found to be randomly polarized on flat PDMS substrates (*S*_*control*_ = *S*_*SPC*_ = 0.00 ± 0.02) (Fig. S2A), but after nearly 8 hours of uniaxial cyclic substrate strain (2 Hz, 8%), a large percentage of the cells had realigned in a direction perpendicular to the strain, mirroring the previous alignment of keratin fibers (Fig. 3A).

To investigate the effect of SPC treatment on cell morphology restructuring, the frequency of cyclic substrate strain was varied between 0.05 and 2 Hz, both with and without SPC treatment, and cell orientation was analyzed after 9 hours. In general, as the frequency increased above a threshold of 0.2 Hz, SPC-treated cells were able to realign perpendicular to the direction of strain more efficiently than the untreated cells (Fig. 3B). Thus, keratin network phosphorylation and subsequent restructuring plays a role in force-induced cell orientation by increasing the degree of morphological adaptation in response to periodic substrate strain.

To understand if cellular keratin restructuring affects the dynamics of this reorientation, the four frequencies of cyclic strain found to induce significantly different orientation parameters in combination with SPC treatment (0.2, 0.5, 1.0, and 2.0 Hz) were applied to untreated and SPC-treated cells, and orientation parameters were calculated at 22 time points over five hours, at which time each cell population had reached an orientation equilibrium. The time constant τ (time to reach 63% of the equilibrium value) was then calculated based on these curves using equation 2 (Fig. 3C). Regardless of SPC treatment, τ decreases as the frequency increases, indicating that higher frequencies stimulate faster cell realignment. SPC treatment is capable of augmenting this effect, but only up to a point; at 2.0 Hz the time constant is equivalent for untreated and SPC-treated populations. We can infer from this that keratin reorganization provides a degree of responsiveness to the cells that can be saturated at high stretch frequencies- it is possible that this level of strain may partially induce defects in the keratin network and thus show no further effect^57^. The general observation that an intact keratin network in the cell periphery slows down actin-driven cell reorganization provides evidence that keratin may function as a type of cytoskeletal dashpot, providing viscoelastic steric hindrance to the more reactive actin network that has been shown to drive cell reorganization^58^.

### Cyclic strain influences Panc-1 cell migration

In addition to cell orientation, the migratory properties of the Panc-1 cells could be calculated during the stretching experiments. This complements the orientation analysis, which focused on cell spanning cytoskeletal networks, by considering the cyclic strain effect on the emergence of newly polymerizing lamellipodia-like networks. Cells seeded on flat control substrates without any strain moved randomly with no preferred direction (Fig. S2A), while on flat control substrates being stretched in the X-direction, cells exhibited biased migration along the Y-axis, perpendicular to the direction of pull (Fig. 3D).

To characterize this behavior, the orientation parameter S_M_ was calculated using the angle between the cell’s migration vector and the direction of strain (the X-axis) (Fig. S1B). S_M_ was then calculated for each strain frequency f, both with and without SPC treatment. A decrease in S_M_ can be observed with an increase in frequency f, which corresponds to an increase in the number of cells migrating perpendicular to the direction of strain. This effect is enhanced in cells treated with SPC, but only for frequencies of 0.5 Hz and greater. A decrease in S_M_ at high frequencies as a result of SPC treatment was observed, with the frequency required for the cells to reach the arbitrary migratory orientation value S_M_ = −0.37 halving from 2 Hz to 1 Hz (Fig. 3E).

Additionally, the average velocity of Panc-1 cells exposed to different strain frequencies was determined. A large distribution of cellular velocities was observed, with the average speeds of the fastest cells as much as sixfold higher than the slowest cells. In many cases, the average speed of cells on stretched substrates was found to not be significantly different from those on unstretched surfaces. Thus, the change in migration vector orientation was likely not a function of any changes in migration speed (Fig. 3F), as no systemic effect of the stretching on speed was observed. On cells treated with SPC, average cell velocity was significantly higher for most stretching conditions, including the unstretched control. This is consistent with our hypothesis that the restructuring of the keratin network provides a less hindered environment for the cell migration-driving actin network to function more efficiently, with the output in this case being an increase in cytoskeleton-generated velocity.

### Panc-1 cell migration on grooved substrates is dependent on keratin phosphorylation and groove depth

**Figure 4:**
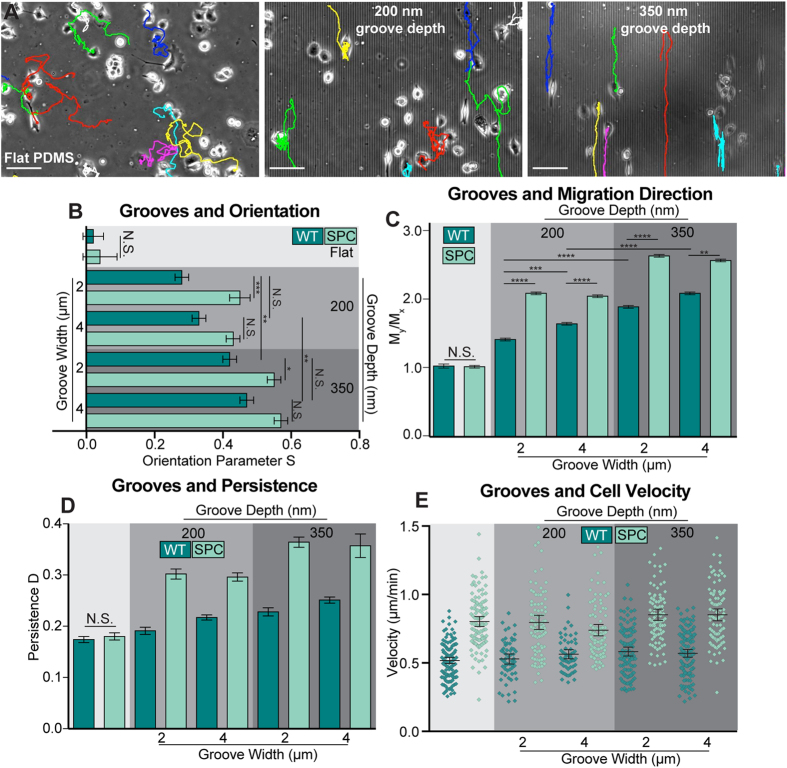
Keratin Phosphorylation and Groove Depth Induce Alignment and Directed Migration. Images of cells on flat control substrates and grooved substrates with different dimensions were obtained, with deeper grooves causing stronger alignment and directed migration (**A**). Analysis of the cells on the grooved substrates revealed that groove width was not as important as groove depth for cell orientation (**B**), cell migration vector orientation (**C**), or migration persistence (**D**). While keratin phosphorylation caused an increase in cell velocity, substrate grooves did not alter the velocity profile of either the untreated or SPC treated cells (**E**). Cell speed increases as a result of SPC treatment were statistically significant (p < 0.0001) by one-way ANOVA with Tukey post hoc tests for all culture conditions. Scale bars = 150 μm (**A**). WT = Untreated (**B**–**E**).

To examine the relationship between contact guidance and keratin network organization, untreated and SPC-treated Panc-1 cells were grown on substrates with and without grooves. On grooved substrates, immunofluorescence staining revealed that the actin network tended to show groove direction-dependent patterns, while the keratin network did not respond as noticeably (Fig. S3). In general, cells plated on grooved substrates tended to align and migrate along the grooves (S > 0), as opposed to the random alignment and movement on flat control substrates (S ≈ 0) (Fig. 4A,B). The angular distributions of untreated and SPC-treated cell migration were plotted in the first quadrant (Fig. S2B), illustrating this bias in migration direction. Furthermore, a decrease in x-axis directed migration is seen in SPC-treated cells on grooved substrates. Thus, the microstructured surface can affect the migration direction of Panc-1 cells, and this effect is enhanced by destabilization of the keratin network. This could potentially be due to an increased degree of cell body deformation into and around the physical microenvironment as a result of the reduction in the stiff keratin network in the periphery of the cell.

By altering the depth or width of the grooves, several important observations could be made. First, the Panc-1 cells aligned along the grooves for all substrates, regardless of the groove dimensions or SPC treatment (Figs 4A,B, S2B). SPC treatment did result in an increased alignment along the grooves for all tested dimensions. Increasing the depth of the grooves from 200 nm to 350 nm and increasing the width of the grooves from 2 μm to 4 μm were both sufficient to increase alignment, with the biggest effect coming from increasing depth. SPC treated cells were also more aligned on deeper grooves, but the groove width and spacing had no effect on SPC treated cells (Figs 4B, S2B).

On control substrates, the ratio of Y-axis movement to X-axis movement is equivalent to 1, indicating unbiased migration. For untreated cells, this ratio increases with increasing groove width and depth, indicating migration parallel to the groove structures. SPC treatment results in further increases in M_y_/M_x_, but again, among SPC-treated populations, the only physical parameter that resulted in different migration characteristics was groove depth (Fig. 4C). Increases in groove depth and width also spurred an increase in directional persistence, indicating smoother, more biased migration, while SPC-treated populations were only affected by increases in groove depth and unaffected by groove width dimensions, although migration persistence was higher across the board in treated cells compared to untreated ones (Fig. 4D). Interestingly, groove dimensions played no role in cell velocity, as all conditions resulted in untreated cell velocities of approximately 0.5 μm/minute. The same was true for the SPC-treated populations, which are generally faster than untreated cells on control substrates. Their velocity was independent of groove dimensions at approximately 0.75 μm/minute (Fig. 4E). It is of note that these observed velocities were statistically similar to values obtained on flat control PDMS surfaces, indicating that grooved substrates do not alter the inherent cell velocity of Panc-1 cells.

Altogether, it is clear that the grooved substrates are capable of biasing untreated cell alignment, migration direction, and directional persistence, and that this effect is amplified by both deeper and wider grooves, as opposed to SPC-treated populations that are only affected by groove depth. Despite the fact that SPC treatment did not play a role in the mechanotransduction of wider grooves, the response to groove depth was generally stronger than in untreated cells, suggesting improvements in contact guidance. For the treated cells, this contact guidance enhancement can be explained by noting that the cells are mechanically softer as a result of the loss of an intact keratin network, allowing them to physically adapt better the grooves or other microscale features. To see if other microscale features also effect differences in contact guidance, cells were next analyzed on PDMS micropillars.

### Migration on pillar substrates

**Figure 5:**
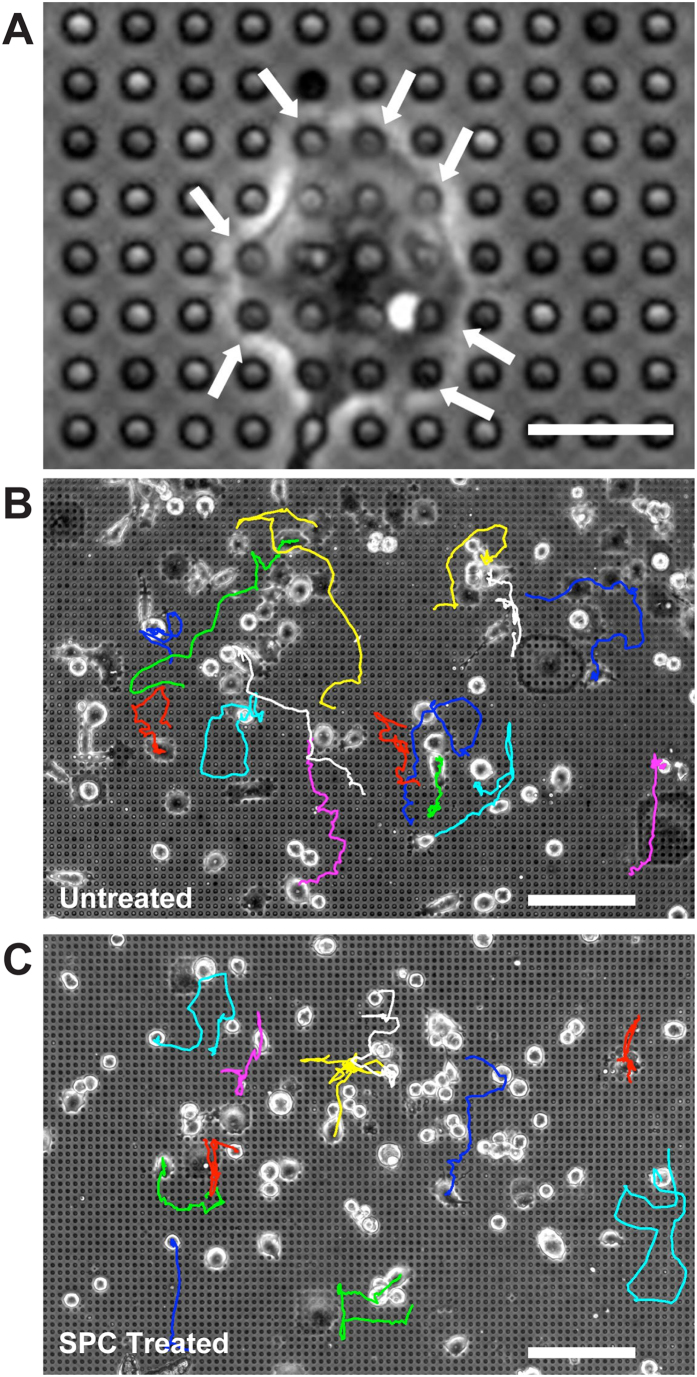
Keratin Phosphorylation and Influences Cell Migration on Pillar Substrates. Panc-1 cells were plated onto substrates containing ECM protein atop pillars, confining adhesion to these points (**A**). Tracking cell migration over a period of eight hours revealed distinct migration patterns in (**B**) untreated and (**C**) SPC-treated cells. Scale bars = 30 μm (**A**), 150 μm (**B**,**C**).

As an alternative to the grooved structures, pillar substrates were used in order to present a micro-structured surface to the cells with a different type of anisotropy. In contrast to the flat control PDMS and the grooved substrates, the fibronectin was only added to the tops of the pillars. As a result, cells could only adhere to the pillar tops via focal adhesions (any cells moving between the pillars were excluded from analysis). This results in a cell morphology in which the periphery is supported along the tops of the columns (Fig. 5A).

The average velocity of cells on the pillars was calculated to be 0.45 ± 0.02 μm/minute, not significantly different from velocity on control substrates. SPC treatment increased the speed of the cells to 0.58 ± 0.02 μm/minute, which was a lower increase than that observed on control and grooved substrates.

Trajectories of untreated and SPC-treated cells migrating on pillars with 4 μm diameters, 8.7 μm heights, and 6 μm spacings were calculated (Fig. 5B,C). The difference between untreated and SPC-treated cells is smaller than the differences observed on grooved substrates, but close inspection reveals that SPC-treated cells tend to prefer movement in either the X- or Y-axis. To investigate this, frequency histograms of the migration trajectories of untreated and SPC-treated cells were constructed (Fig. S2C)^59^. The untreated plot shows the characteristic flat distribution expected for randomly migrating cells, but the SPC-treated plot shows distinct peaks in both the vertical Y-axis direction and the horizontal X-axis direction, indicating a clear orthogonal bias in migration. This corresponds to the axes of symmetry in the pillared substrates leading to the conclusion that keratin phosphorylation is capable of enhancing the mechanosensitivity of migrating cancer cells, as on the pillars the X- and Y-axis directions correspond to the closest physical signal available to the cells.

For both the untreated and SPC-treated cell populations, the average orientation parameter S was 0.00 ± 0.04, indicating random cell alignment. This result appears to be at odds with the orthogonally aligned results from SPC-treated cells shown in Fig. S2C, but this is likely due to preferred alignment in the X or Y direction being cancelled out. This is similar to the calculation of the M_y_/M_x_ parameter, which was found to be 1.01 ± 0.04 for untreated cells and 1.02 ± 0.06 for SPC-treated cells. Overall, treatment of Panc-1 cells with SPC and the resultant keratin reorganization can influence the migration dynamics of Panc-1 cells on pillars in a similar manner to that found on grooved substrates.

## Conclusions

In general, cells with less keratin expression have been shown to be softer and more susceptible to deformation^16^. Furthermore, invasive cancer cells have been found to be much softer than both healthy cells and non-invasive cancer cells^60^,^61^. In many cases, this loss of stiffness has been analyzed in the context of three-dimensional cell migration and invasion^16^,^60^. While the migratory mechanisms underlying three-dimensional invasion are in many ways distinct from those guiding two-dimensional migration, there is evidence that nanoscale topography of two dimensional substrates is correlated with alterations in cell stiffness^62^. As the interactions between nanoscale substrate conditions and cancer cells has been shown to play a role in cancer treatment *in vivo*^63^, an elucidation of this link *in vitro* is important. We have shown that in Panc-1 pancreatic cancer cells, the phosphorylation of keratin leads to perinuclear reorganization, in agreement with previous work^64^. Our observation that Panc-1 cells align perpendicularly to cyclic substrate stretch is in line with previous observations in other cell types^57^, but in contrast to a study on tumor capillary endothelial cells, which showed reduced perpendicular rearrangement after exposure to a 1 Hz, 10% uniaxial strain^65^. This may be due to differences in tissue origin; it is intriguing to wonder whether and how SPC treatment might play a role in stretch-responsive orientation in other cancer cell lines similar to the effect seen in Panc-1 cells. Ultimately, this effect may be due to the effects of steric hindrance. As the keratin network (and likely the vimentin network, based on recently published studies^32^ and the data in Fig. 1G) becomes destabilized and reorganizes around the nucleus, the cell volume in the periphery becomes less entangled. This reduction in steric hindrance or entanglement may then be enough to generally unencumber the actin cytoskeleton, which has been shown in many studies to be a key player if not the main effector of a wide variety of cytoskeleton-based characteristics, including cell orientation, migration, and mechanotransduction^66^,^67^,^68^.

## Materials and Methods

### Cell Culture

All chemicals were purchased from Life Technologies (Darmstadt, Germany). Human pancreatic cancer cells (Panc-1) (ECACC #87092802) were used for all cell experiments due to their previous keratin cytoskeleton characterization^33^. Cells were cultured at 37 °C and 5% CO_2_ in 75 cm^2^ (T-75) flasks in a media composed of Dulbecco’s Modified Eagle Medium (DMEM) 11960 supplemented with 10% (vol/vol) fetal bovine serum (FBS) (PAA Laboratories, Cölbe, Germany), 1% (vol/vol) penicillin/streptavidin (Pen/Strep), and 1% (vol/vol) L-glutamine. Media was replaced every 2–3 days, and cells were passaged with 2.5% trypsin-EDTA upon reaching 70–80% confluency. Chemical inhibitor concentrations for Cytochalasin D (1 μM), Nocodazol (3 μM), and SPC (10 μM) were used in accordance with reported literature values^34^,^69^,^70^.

### Sample Preparation

PDMS substrates were cleaned in 70% ethanol followed by PBS for 5 minutes each. The substrate surface was then coated with 50 μg/mL human fibronectin (Calbiochem #341635, Germany) for two hours, followed by two PBS rinses. Panc-1 cells were then seeded at a concentration of 80 cells/mm^2^ and stored overnight in an incubator. One hour prior to the start of an experiment, the substrate was mounted, leveled, and allowed to equilibrate in experimental media (5% FBS) in an incubating chamber and the cells were also placed in experimental media. For inhibitor experiments, the experimental media was dosed to a concentration of 10 μΜ.

### Immunocytochemistry

Cell populations selected for IF staining were rinsed with PBS and incubated with a preheated 4% (wt/vol) paraformaldehyde (PFA) fixative solution in PBS for 10 minutes at 37 °C. Cells were then rinsed three times in PBS for 8 minutes, followed by permeabilization in 0.5% (vol/vol) Triton X-100 (Carl Roth, Karlsruhe, Germany) in PBS for 15 minutes. After another two PBS rinses, cells were blocked for three hours at room temperature with 0.2% (vol/vol) fish gelatin in PBS (Sigma) to prevent non-specific binding. For immunostaining of keratin, the mouse anti-pan-cytokeratin antibody (KL1, Immunotech SAS/Beckman Coulter, Marseille, France) was used at a 1:200 dilution in 0.5% Triton X-100/0.2% fish gelatin. After rinsing in PBS, the cells were stained with the following solution for one hour: AlexaFluor 488 chicken anti-mouse (1:250), AlexaFluor 568 Phalloidin (1:50), and Hoechst 33342 (1:1000) diluted into 0.5% Triton X-100/0.2% fish gelatin in PBS. The stained cells were then mounted with Aqua-Poly/Mount Media (Polysciences, Eppelheim, Germany) onto a glass slide.

### Microscopy

Phase contrast images were obtained with a Zeiss Axiovert 200 M microscope used in conjunction with four different lenses (5X A-Plan 0.12 Ph0, 10X A-Plan 0.25 Ph1, 20X EC Plan-Neofluar 0.50 Ph2, and 40X LD A-Plan 0.50 Ph2), an X-Cite 120 W DC metal halide lamp (EXFO Europe, England), a microscope-mounted cell culture chamber (EMBL, Heidelberg, Germany) maintaining constant culture conditions of 37 °C and 5% CO_2_, and an AxioCam MR CCD camera (Zeiss).

Fluorescence images were obtained on both a Zeiss AxioObserver Z1 transmitted light microscope and an Imager Z1 upright microscope, both used in conjunction with 3 different lenses (20X LD Plan-Neofluar 0.40 Ph2 Corr., 40X LD Plan-Neofluar 0.60 Corr., and 63X LD Plan-Neofluar 0.75 Corr.), a Colibri fluroescent lamp with 365, 470, 505, and 590 nm LED modules and the corresponding filters (60HE, 62HE) (Zeiss), and a cell culture chamber.

Additional images were taken with a scanning electron microscope (SEM) (Ultra-55 Field Emission SEM, Gemini, Zeiss, Germany) and a white light interferometer (WLI) (Zygo NV5000, Middlefield, CT).

### Cell Substrate Stretching

Cell substrate stretching was performed using a stretching device developed and elaborated on in previous works^57^, consisting of a DC servo motor (Faulhaber, Schöneich, Germany) which can apply a periodic stretch to clamped PDMS substrates along a single axis. The stretching device and microscope were controlled by an integrated, custom-built Visual Basic program (Microsoft, USA) in AxioVision software. The stretch frequencies used were 0.05, 0.1, 0.2, 0.5, 1.0, and 2.0 Hz with a constant amplitude of 8%. Three independent experiments were performed for each frequency setting, each with a duration of 8 hours.

### Morphology Analysis

Time-lapse phase contrast images were used for cell morphology analysis in ImageJ (NIH). Metrics calculated were cell orientation, a time constant representing the response to strain over time, and cell area. For each metric, multiple cells from three independent experiments were averaged. The orientation parameter S was calculated from outlines of each cell manually drawn in ImageJ and corresponding fitted ellipses. The angle between the major axis of the ellipse and the direction of substrate stretching was then calculated. In control experiments, the X-axis was used as a reference direction. The average of the orientation of cells in response to the cyclic strain of the substrates was quantified by the non-polar orientation parameter *S* in equation 1^57^,^59^. The angular distribution function *f(φ*) and the orientation angle *φ* are given by the Fokker-Planck equation^71^ from which a order parameter can be calculated.


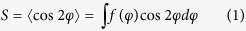


The orientation parameter *S*_*i*_ was determined for each individual cell, and the average of all orientations *S* for each time point was determined 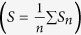, where n is the number of cells analyzed. The characteristic time τ describes the time it takes for a population of cells to proceed from their initial average orientation 〈cos 2*φ*〉_0_ to 63% (1/*e*) of their final average orientation 〈cos 2*φ*〉_*Max*_. To calculate this, the time course of the mean value of the orientation parameter *S* (equation 1) was plotted and fitted in Origin 6.0 by equation 2.


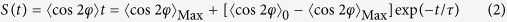


For each experiment, an average τ value was calculated. The cell area A was determined by manually outlining cells in ImageJ and calculating the enclosed area. This value was obtained for multiple cells at each time point during long-term experiments, and then normalized to the initial area value.

### Cell Migration

Cell migration was assessed in ImageJ using phase contrast images from time points *t*_*i*_ with the manual tracking plugin to track the center of each cell, with all analyzed cells followed for at least five hours. Speed and direction of migration were obtained in Microsoft Excel, and the Chemotaxis and Migration Tool 1.01 ImageJ application (Integrated BioDiagnostic, Martinsried, Germany) was used to analyze cell migration on grooves and pillar substrates, with the Directionality D of migration determined as shown in equation 3.


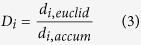


Here, *d*_*i,euclid*_ represents the overall distance that the cell has covered, while *d*_*i,accum*_ represents the cumulative sum of distance from each individual step. Time-lapse images were also used to measure the angular distributions of cell migration, which were then organized in histograms.

For cell speed calculations, the average speed of each cell was calculated by averaging all instantaneous speeds found at each time point and displayed in Figs 3F and 4E. For experiments with different substrate stretch frequencies, data was collected from between 2 and 4 individual experiments, with 17,573 instantaneous speed measurements performed over 758 cells altogether. For experiments with different grate dimensions, data was collected from between 2 and 4 individual experiments, with 96,324 instantaneous speed measurements performed over 742 cells altogether.

Analogous to the metric of chemotactic index^45^, a parameter measuring the cell migration projected onto two perpendicular axes was calculated. For this study, the X-axis corresponded to the direction of pull in strain experiments, while the Y-axis corresponded to the direction of the grooves in the grooved substrates. In control experiments, the X-axis corresponded to the arbitrary axis. The movements of a given cell along these axes were determined using equation 4 for every migration step, allowing for the ratio (*M*_*y*_/*M*_*x*_)_*i*_ to be calculated. A mean value for each cell could be calculated by averaging the ratio from each time point, shown here as 
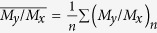
. Finally, a mean value can be obtained for each condition by averaging the ratio from each cell, shown here as 
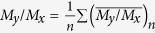
.


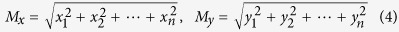


### Actin and Keratin Orientation

The orientation of actin and keratin networks was observed by both ICC staining and EM images and processed with custom ImageJ macros. A Single Alignment Macro (SAM) analyzed the orientation of actin or keratin networks by first dividing a single channel fluorescence image into sub-areas with dimensions of 32 × 32 pixels. Following this, the overall orientation of the texture in the subarea was determined by measuring the moment of inertia in the fast Fourier transform subimage^72^. As a result, the orientation and corresponding angle υ of the filaments with reference to the x-axis in the sub-areas were determined and graphically displayed (Fig. 2B). The mean 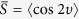 of all the filaments in all the sub-areas revealed the average orientation of the network in the cell. The results shown were calculated from the average of all investigated cells 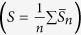, where n is the number of cells analyzed. A Double Alignment Macro (DAM) was used to analyze the orientation of actin and keratin networks in reference to each other using an ICC image with two channels corresponding to the two networks. If a sub-area has both actin and keratin networks, orientation angles υ_actin_ and υ_keratin_ are determined and then analyzed with Microsoft Excel’s Autocorrelation Function, yielding a paired correlation coefficient *r*. Final results 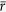 are based on the average of *r* over multiple cells.

### Photolithography

Photolithography with S1818 positive photoresist was utilized to produce the groove and pillar structures. A 3-inch p-type silicon wafer (100 orientation, Siegert Consulting, Aachen, Germany) was placed in Piranha solution (3:1 sulfuric acid to 30% hydrogen peroxide) for 2 hours. Next, the wafer was rinsed in acetone and isopropanol for three minutes each, followed by a rinse in ultrapure water and heating for 10 minutes on a 200 °C hotplate. The wafer was then centered on a spin coater (WS-400B-6NPP-Lite, Laurell Technologies Corp., USA) and blown dry with a nitrogen gun. 3 mL of S1818 positive photoresist were added to the center of the wafer, and the resist was spread in a two step spinning program (500 rpm for 5 seconds, followed by 3000 rpm for 30 seconds) optimized to obtain a desired photoresist thickness of approximately 1.8 μm. Excess solvent was then evaporated by heating at 115 °C for one minute, and the wafer stored at room temperature for five hours. Alignment with a micropatterned chrome photomask (ML&C, Jena, Germany) displaying either 1 cm^2^ fields of circles of diameter 4 μm and center spacing 10 μm in a square arrangement or 1 cm^2^ fields were filled with long rectangles of 2 or 4 μm width and 2 or 4 μm center spacing was performed in an MJB4 manual mask aligner (Sweet and Microtech, Germany) with an LIF350 filter. Exposure was carried out using a 350 W mercury vapor lamp with a wavelength of 365 nm for two seconds with hard contact wedge error correction (WEC) at 1.7 bar. Areas of photoresist exposed to UV were dissolved and washed away in 30–40 seconds using MF-319 Microposit developer (MicroChem, USA) followed by rinsing with ultrapure water and drying with a nitrogen gun.

### Reactive Ion Etching (RIE)

RIE with the ion etcher Plasmalab 80 Plus (Oxford Instruments, England) was used in conjunction with photolithography to achieve the high aspect ratios associated with micropillars. After the photolithography-patterned mask was placed inside the etcher, the chamber was cooled to −20 °C to increase etching selectivity. The etching process consisted of 4 sub-steps constituting one cycle. After each etching and passivation step, the chamber was pumped with a vacuum to eliminate waste products. Table 1 contains the complete steps and parameters for the entire etch process. A mixture of SF_6_ and HCF_3_ at a ratio of 5:6 was used as the etching gas, and during passivation steps only HCF_3_ was introduced into the chamber. The photoresist-covered Si wafers were subjected to 150 cycles, providing an etch depth of approximately 8.7 μm. A polyurethane polymer consisting of 66% EBECRYL284 diacrylate prepolymer (Cytec Surface Specialties, Germany), 30% M3160 (MIWON, Korea), 1.5% Irgacure184 and 1.5% Darocur1173 (Ciba Chemicals Specialty, Switzerland) and 1% Rad2200N (Tego Chemie Service, Germany) was then cast to make the final mold. The sample was stored for 30 minutes at room temperature to remove air pockets, then placed in an ultraviolet radiation chamber (UVACube 100, Honle UV Technology, Germany) and irradiated for one minute with UVA light, followed by baking for one hour at 65 °C. Following this, the polyurethane mold was mounted in a metal frame and used as a master mold for the fabrication of PDMS substrates.

### Thermal Chromium Deposition

Following the fabrication of grooved surfaces on silicon wafers with photolithography, thermal deposition with a PVD system (ZWE Thin Film Laborator, MPI, Stuttgart, Germany) was used to coat the wafers with either 200 nm or 250 nm of chromium, dictating the height of the grooves. The chromium coated wafers were then heated for 2 hours at 65 °C to drive voltages in the chromium layer, then placed in a glass dish and covered with S1818G2 developer (MicroChem, USA) and sonicated for one minute, yielding a Si-chromium master mold for use with PDMS.

### Substrate fabrication and characterization

The silicone-based elastomer polydimethylsiloxane (PDMS) (Sylgard 184, Dow Corning, USA) was used as a bulk material at a 10:1 base to crosslinker ratio for the fabrication of the substrates, yielding substrates with a Young’s modulus of approximately 2 MPa. Degassed PDMS was poured into the appropriate molds, with control substrates fabricated against a flat mold, and allowed to polymerize overnight at 65 °C. PDMS substrates containing no features (control), grooves, and pillars were prepared and characterized by white light interferometry (Zygo NewView 5000, Middlefield, CT, USA) as shown (Fig. S4A). The grooves were found to have a width *B* of 4.01 μm ± 30 nm, a center spacing *A* of 6.02 μm ± 40 nm, and a depth *H* of 351 nm ± 5 nm (Fig. S4B). The average height of the pillars was 8.7 μm ± 50 nm, the average width *B* was 4.02 μm ± 40 nm and the distance between the center positions of the pillars *A* was 10.03 μm ± 50 nm (Fig. S4C), confirming that the dimensions of both the column substrates and the groove substrates match the dimensions of the patterned masks used during the photolithography process.

### Statistics

All experiments were performed in triplicate unless otherwise indicated. Error bars shown are the standard error of the mean (s.e.m.), except in Figs 3F and 4E, which display 95% confidence intervals. Significance for single comparisons was assessed by t-test at a significance threshold of p < 0.05 or lower as indicated. Significance for multiple comparisons was assessed with ANOVA and a post-hoc Tukey test at a significance threshold of p < 0.05 or lower as indicated. Values less than 0.1 were noted. For instances where data is not significantly different, N.S. is stated.

## Additional Information

**How to cite this article:** Holle, A. W. *et al*. Intermediate filament reorganization dynamically influences cancer cell alignment and migration. *Sci. Rep.* **7**, 45152; doi: 10.1038/srep45152 (2017).

**Publisher's note:** Springer Nature remains neutral with regard to jurisdictional claims in published maps and institutional affiliations.

## Supplementary Material

Supplementary Figures

## Figures and Tables

**Table 1:** Reactive Ion Etching Settings.

Step	t(s)	p(mTorr)	SF_6_(sccm)	CHF_3_(sccm)	P_RF_(W)	P_ICP_(W)
Etching	5	40	15	18	30	300
Pumping	7	40	0	0	0	0
Passivation	8	70	0	50	30	100
Pumping	7	40	0	0	0	0
