# Supplementary material for: Intermediate filament reorganization dynamically influences cancer cell alignment and migration
Source: Sci Rep. 2017 Mar 24;7:45152. doi: 10.1038/srep45152 (PMC5364536; doi:10.1038/srep45152)
Supplement: Supplementary Figures [file srep45152-s1.pdf]

Title Page- Supplementary Figures

Full Title:

"Intermediate filament reorganization dynamically influences cancer cell alignment and migration"

Short Title:

"Cancer Cell Migration on Microstructures"

Authors:

Andrew W. Holle[1], Melih Kalafat[1], Adria Sales Ramos[1], Thomas Seufferlein[4], Ralf Kemkemer†[1,2*], Joachim P. Spatz†[1,3]

Affiliations:

1. Department of New Materials and Biosystems, Max Planck Institute for Intelligent Systems, Stuttgart, Germany
2. Reutlingen University, Reutlingen, Germany
3. Department of Biophysical Chemistry, University of Heidelberg, Heidelberg, Germany
4. Department of Internal Medicine, University of Ulm, Ulm, Germany

* Corresponding Author: ralf.kemkemer@reutlingen-university.de

SUPPLEMENTARY FIGURE 1

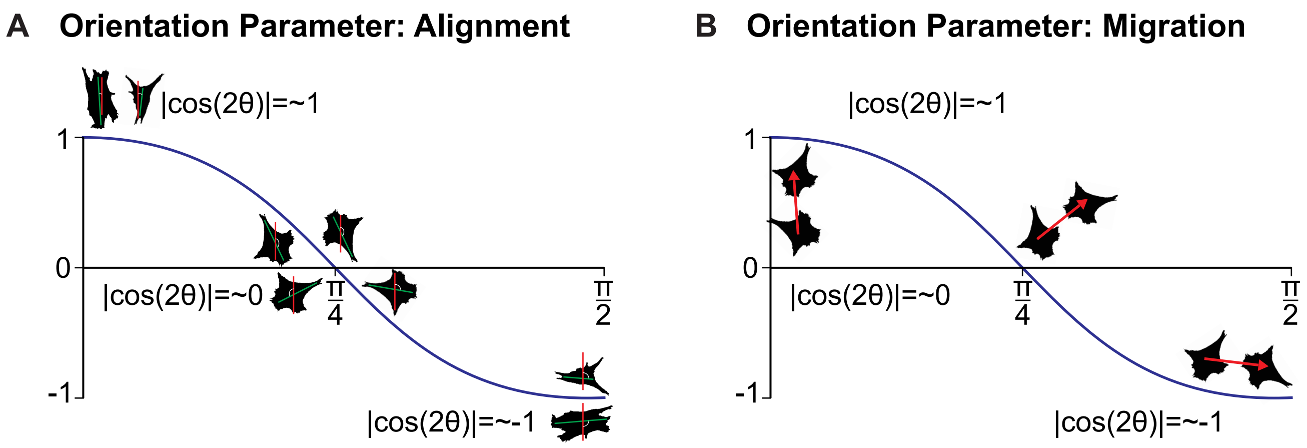

Figure S1: Orientation Parameter Calculation. (A) To determine patterns in cell alignment throughout the population, the major axis of the cell (green) was drawn and compared to a fixed axis (red). The angle between these two lines was then determined and the cosine of twice this angle was calculated to normalize the angle in the first quadrant. (B) To do the same for cell migration, the line connecting the center of mass of a cell between two time points was drawn (red) and the angle between this vector and a fixed axis was calculated and normalized to the first quadrant. For experiments with cyclic strain, the x-axis was the fixed axis, yielding perpendicular orientation and migration that gave orientation parameter values below 0. For experiments with vertical grooves, the y-axis was the fixed axis, yielding parallel orientation and migration that gave orientation parameter values above 0.

SUPPLEMENTARY FIGURE 2

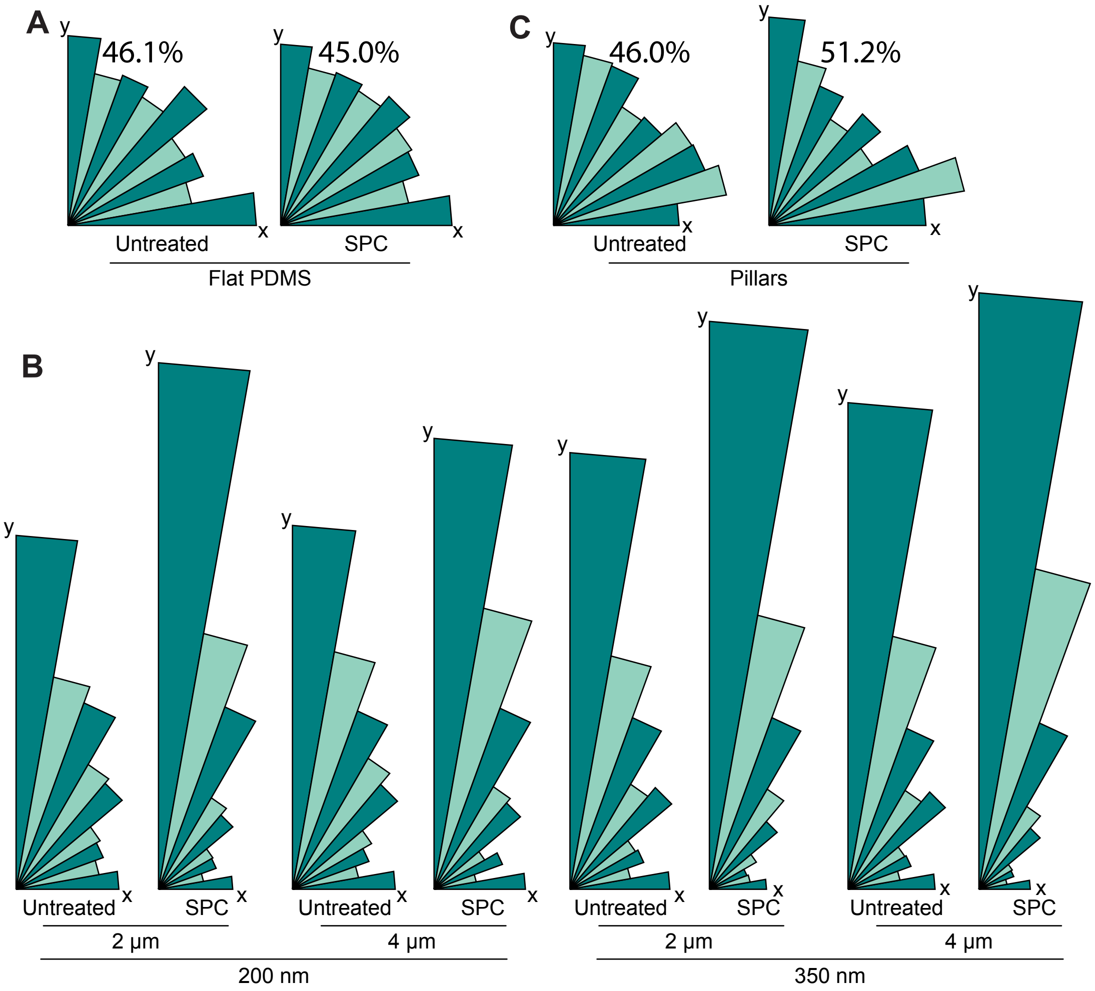

Figure S2: Rose Plots of Migration Vectors in One Quadrant. A) Untreated and SPC-treated cells exhibited no directional bias on flat PDMS control substrates. Percentages of cells migrating in orthogonal directions (0-20 and 70-90 degrees) are displayed above the plot. B) On substrates with grates, migration direction was primarily biased in the y-direction (parallel to the grates), with the strongest bias in migration direction observed in SPC treated cells. 350 nm deep grates were also more effective than 200 nm deep grates in biasing migration direction. C) Untreated cells exhibited unbiased migration on PDMS pillars, but the addition of SPC resulted in a significant bias in the x and y directions, corresponding to the axes of symmetry in the PDMS patters. Percentages of cells migrating in orthogonal directions (0-20 and 70-90 degrees) are displayed above the graph.

SUPPLEMENTARY FIGURE 3

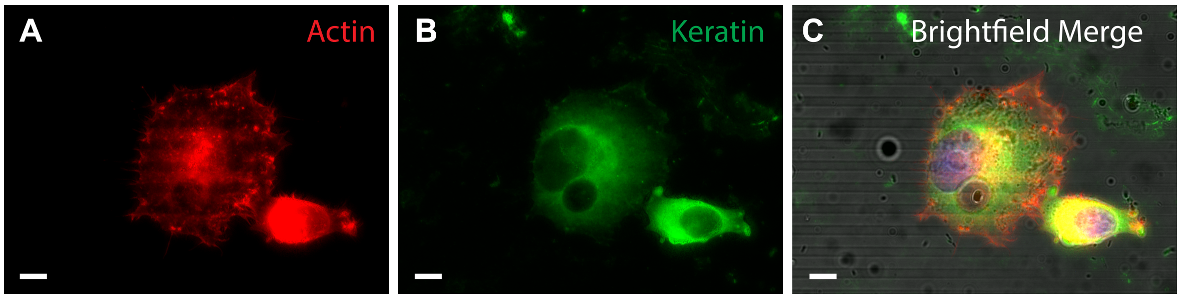

Figure S3: Cytoskeletal Morphology on Grooved Substrates. Representative images of the actin (A) and keratin (B) elements of the cytoskeleton on grooved substrates (C).

SUPPLEMENTARY FIGURE 4

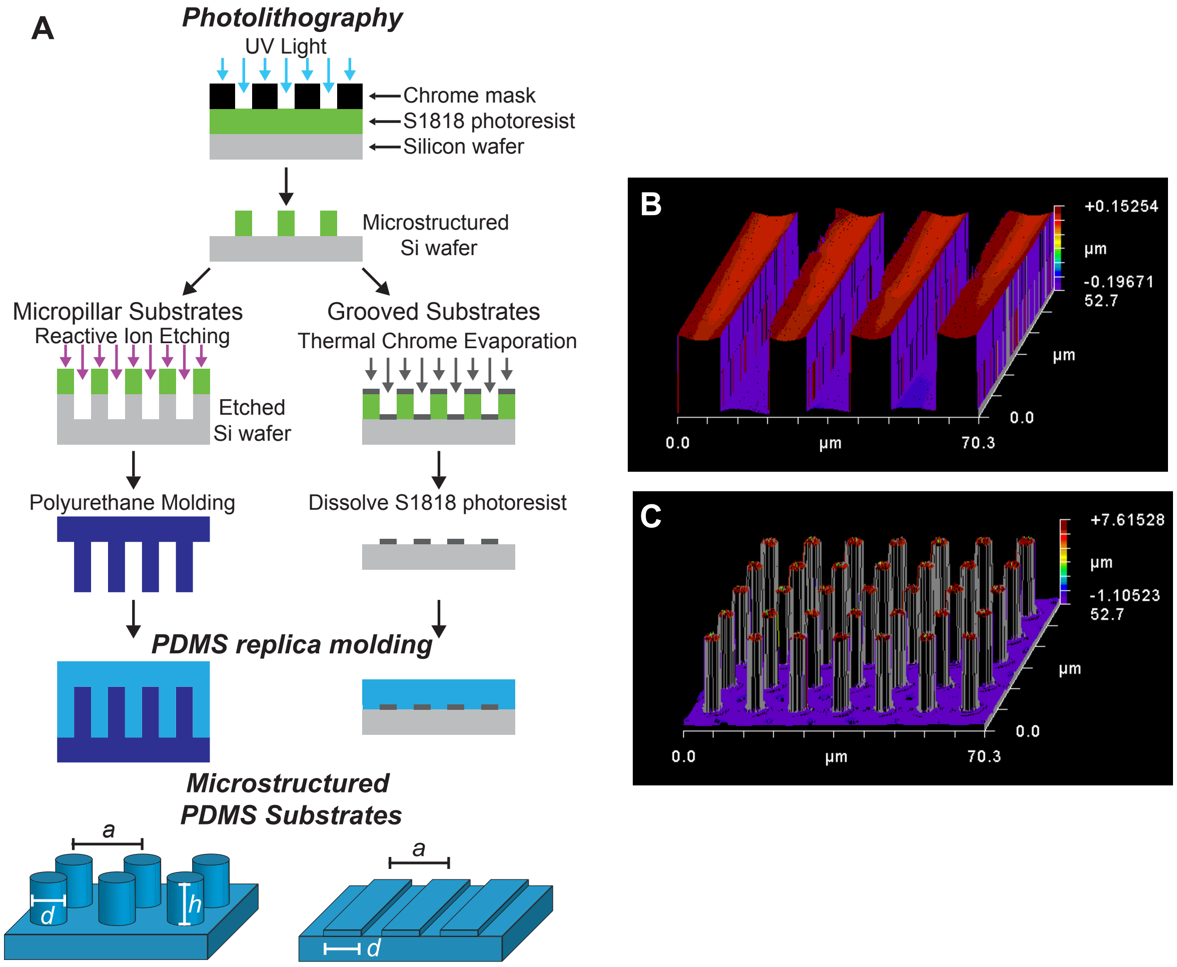

Supplementary Figure 4: Substrate Fabrication and Validation. (A) Photolithography was used for both the grooved and pillared substrates in order to expose desired areas of photoresist to UV light. For grooved substrates with widths d and groove spacing a, the microstructured silicon wafer was exposed to Thermal Chrome Evaporation, followed by photoresist removal and subsequent replica molding. Grooved substrates were evaluated with white light interferometry (B). For micropillar substrates with height h, diameter d, and inter-pillar spacing a, reactive ion etching was used to etch the patterned silicon wafer, followed by polyurethane molding and PDMS replica molding. White light interferometry was also used to evaluate the micropillar structures (C).
